# A multidisciplinary approach to severe bronchopulmonary dysplasia is associated with resolution of pulmonary hypertension

**DOI:** 10.3389/fped.2023.1077422

**Published:** 2023-03-30

**Authors:** Delphine Yung, Emma O. Jackson, Alyssa Blumenfeld, Gregory Redding, Robert DiGeronimo, John K. McGuire, Meredith Riker, William Tressel, Sara Berkelhamer, Laurie C. Eldredge

**Affiliations:** ^1^ Division of Cardiology, Department of Pediatrics, University of Washington School of Medicine, Seattle, WA, United States; ^2^ Heart Center, Seattle Children’s Hospital, Seattle, WA, United States; ^3^ Department of Pediatrics, University of Washington, Seattle, WA, United States; ^4^ Division of Pulmonology, Department of Pediatrics, University of Washington School of Medicine, Seattle, WA, United States; ^5^ Division of Neonatology, Department of Pediatrics, University of Washington School of Medicine, and Seattle Children’s Hospital, Seattle, WA, United States; ^6^ Division of Critical Care Medicine, Department of Pediatrics, University of Washington School of Medicine, Seattle, WA, United States; ^7^ Collaborative Health Studies Coordinating Center, Department of Biostatistics, University of Washington, Seattle, WA, United States

**Keywords:** bronchopulmonary dysplasia, pulmonary hypertension, sildenafil, miltidisciplinary care, neonate

## Abstract

**Objective:**

To describe our multidisciplinary bronchopulmonary dysplasia (BPD) consult team's systematic approach to BPD associated pulmonary hypertension (PH), to report our center outcomes, and to evaluate clinical associations with outcomes.

**Study design:**

Retrospective cohort of 60 patients with BPD-PH who were referred to the Seattle Children's Hospital BPD team from 2018 to 2020. Patients with critical congenital heart disease were excluded. Demographics, comorbidities, treatments, closure of hemodynamically relevant intracardiac shunts, and clinical outcomes including time to BPD-PH resolution were reviewed.

**Results:**

Median gestational age of the 60 patients was 25 weeks (IQR: 24–26). 20% were small for gestational age (SGA), 65% were male, and 25% received a tracheostomy. With aggressive cardiopulmonary management including respiratory support optimization, patent ductus arteriosus (PDA) and atrial septal defect (ASD) closure (40% PDA, 5% ASD, 3% both), and limited use of pulmonary vasodilators (8%), all infants demonstrated resolution of PH during the follow-up period, including three (5%) who later died from non-BPD-PH morbidities. Neither SGA status nor the timing of PH diagnosis (<36 vs. ≥36 weeks PMA) impacted the time to BPD-PH resolution in our cohort [median 72 days (IQR 30.5–166.5)].

**Conclusion:**

Our multidisciplinary, systematic approach to BPD-PH management was associated with complete resolution of PH with lower mortality despite less sildenafil use than reported in comparable cohorts. Unique features of our approach included aggressive PDA and ASD device closure and rare initiation of sildenafil only after lack of BPD-PH improvement with respiratory support optimization and diagnostic confirmation by cardiac catheterization.

## 1. Introduction

Bronchopulmonary dysplasia (BPD) is the most common chronic lung disease of prematurity and rates are increasing with improved survival of extremely premature infants (1, 2). BPD is defined in infants born at less than 32 weeks gestation as an oxygen and/or respiratory support requirement after 36 weeks postmenstrual age (PMA) (3–6). Pulmonary hypertension (PH) associated with BPD (BPD-PH) is an important comorbidity and 2.7 times more common in infants with severe BPD (7). Infants with BPD-PH have four times greater mortality risk than those with BPD alone, with estimates ranging from 21% to 50% (8–10). BPD-PH is also associated with increased risks of tracheostomy, feeding impairment, home oxygen use, hospital readmission, reduced neurodevelopmental outcome, and poor growth (11).

The approach to managing BPD-PH varies widely, including screening, diagnosis, treatment, use of cardiac catheterization, and pulmonary vasodilator therapy. Current guidelines recommend management of BPD-PH by a multidisciplinary PH team and initiation of sildenafil if PH remains after optimization of cardiac and respiratory disease management (12–15). Sildenafil has become a common treatment for patients with BPD-PH, and despite recommendations for cardiac catheterization prior to initiation of sildenafil (16), fewer diagnostic catheterizations are being performed (13, 17). However, the benefits of sildenafil remain unclear, as a meta-analysis including 101 patients concluded that sildenafil use in BPD-PH may be associated with improvement in pulmonary pressure and respiratory scores, but not mortality (18).

To improve care for BPD patients in the level IV neonatal intensive care unit (NICU) at Seattle Children's Hospital (SCH), a multidisciplinary inpatient BPD consult team was formed in 2017. Weekly consults were performed using a standardized approach to management of BPD-PH. Our team leveraged the synergistic expertise of all team members and prioritized optimization of respiratory support and treatment of comorbidities before initiation of PH medication. We now report the outcomes of the multidisciplinary systematic approach to BPD-PH at our center.

## 2. Methods

### 2.1. Patients and study design

The multidisciplinary BPD team consults on patients in our Level IV NICU who are >36 weeks PMA with severe BPD, or earlier at neonatology request for patients with evolving severe BPD. Referred patients are followed throughout their hospital course until discharge. All patients are out born and transferred due to need for pediatric subspecialty care, such as surgery or need for complex consultative care. We retrospectively reviewed records of all infants followed by the SCH BPD team during 2018–2020 for BPD-PH.

### 2.2. Management strategy for BPD-PH

**Figure 1:**
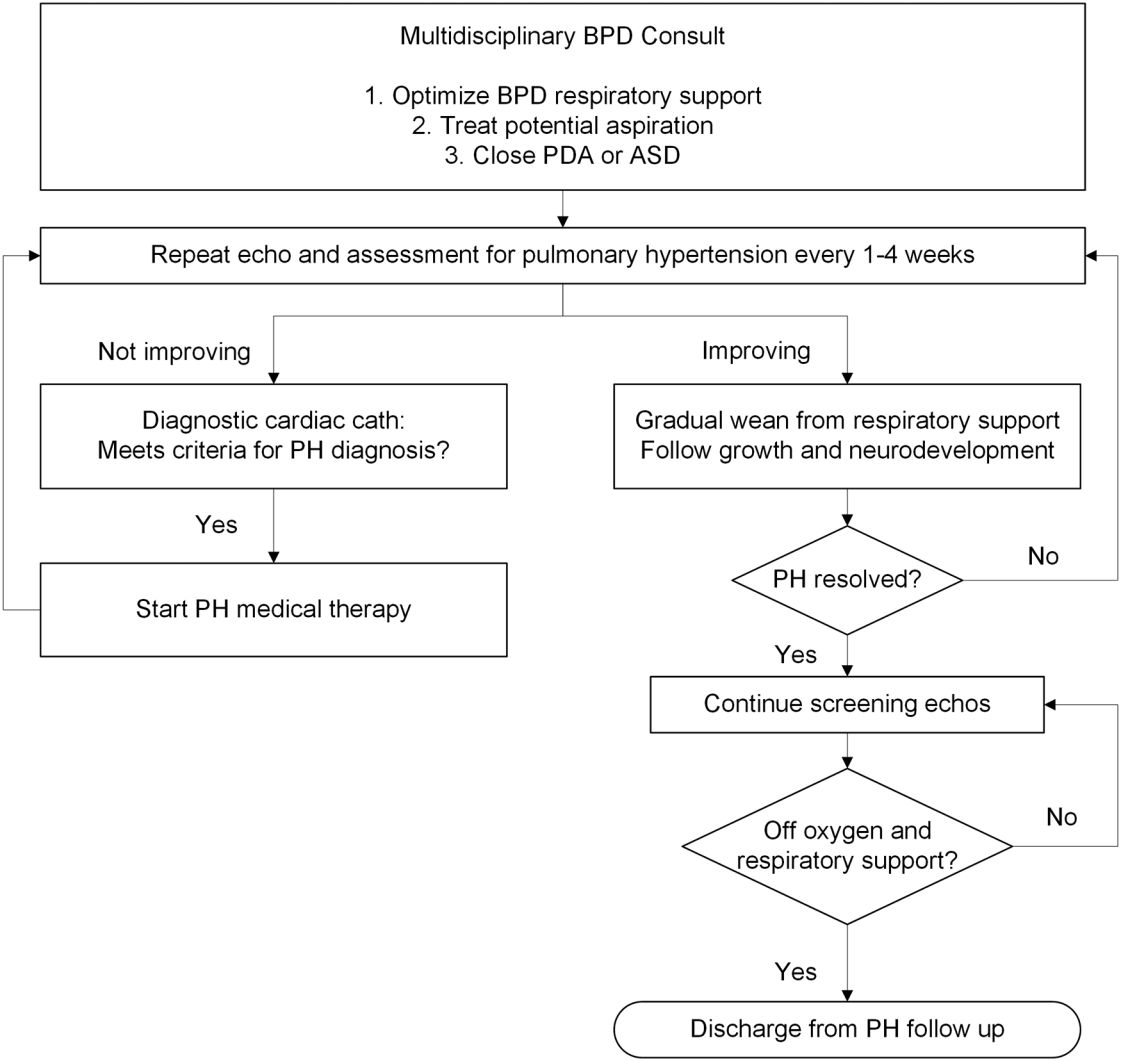
Algorithm of care for bronchopulmonary dysplasia-pulmonary hypertension (BPD-PH). PH, pulmonary hypertension; PDA, patent ductus arteriosus; ASD, atrial septal defect; Cath, catheterization; Echo, echocardiogram.

The multidisciplinary inpatient BPD team was made up of pediatric specialists from neonatology, pulmonology, cardiology, critical care, respiratory therapy, feeding therapy, developmental therapy, and nutrition. Team members met weekly with the primary service teams to systematically review clinical events and trends, and to establish collaborative care plans. Screening and treatment for concomitant PH was discussed, including PH-specialist interpretation of the most recent echocardiogram. PH diagnosed in patients before 36 weeks PMA was considered “evolving” BPD-PH. Figure 1 shows the algorithm for managing BPD-PH.

#### 2.2.1. Optimization of respiratory support

Respiratory course was reviewed at each meeting, including symptoms, respiratory support, respiratory rates, oxy-hemoglobin saturations, chest imaging, laboratory values, and medications. Other data reviewed included length and weight trajectories, feeding tolerance, developmental progress, and rate of overall improvement. If BPD-PH was diagnosed, BPD respiratory support strategies (invasive mechanical ventilation, noninvasive positive pressure ventilation, continuous positive airway pressure, high flow nasal cannula) were optimized to minimize work of breathing, avoid hypercapnia, decrease hyperinflation, and support optimal growth and development consistent with strategies described by the BPD collaborative (4). As a team, we implemented the high tidal volume (10–14 ml/kg), low rate (16–20 breaths per minute), and long inspiratory time (0.5–0.7 s) ventilator strategy published by Abman et al., recognizing the need for unique ventilator management due to high airway resistance in severe established BPD. PEEP was clinically titrated to the individual patients' physiology based on the presence or absence of bronchoscopy-determined central airway malacia, severity of dynamic lower airway obstruction, and frequent bedside assessment of ventilator synchrony. In patients with frequent oxygen desaturations <94% despite bedside titration to goal, a minimum supplemental oxygen level (for example 0.25–0.3 FiO_2_) was considered, but carefully balanced and frequently reassessed to avoid hyperoxia. Trials of systemic and inhaled steroids, diuretics, and inhaled bronchodilators were considered on a case-by-case basis. Chest computed tomography (CT) imaging of lung parenchyma and pulmonary veins, and airway endoscopy were obtained for cases with disproportionate hypoxia or hypercapnia, or persistent evidence of BPD-PH by echocardiogram despite optimization of respiratory support. Tracheostomy was considered for chronic respiratory failure requiring long term invasive ventilation. Optimization of respiratory support continued until the patient stabilized, growth normalized, neurodevelopmental progress was established, and BPD-PH improved.

#### 2.2.2. Treatment of suspected aspiration

Patients with BPD-PH taking any oral feeds underwent clinical swallow evaluation and, if recommended, video fluoroscopic swallowing study. The feeding method was adjusted per feeding therapy and BPD team recommendations. Nasogastric feeding tubes were placed if needed to decrease aspiration risk and were changed from gastric to post-pyloric with concerns for significant reflux or aspiration, lack of respiratory status improvement, or worsening BPD-PH. Gastric feeds volumes and rate of delivery were adjusted to optimize feeding tolerance and minimize aspiration risk. Nissen fundoplication is not routinely performed at SCH, so all post-pyloric feeds were *via* nasoduodenal or gastrojejunal tubes.

#### 2.2.3. Echocardiogram

PH was defined as tricuspid regurgitation jet > 2.5 m/s, interventricular septum flattened in systole, or bidirectional shunting in the presence of a patent ductus arteriosus (PDA) or ventricular septal defect. All echocardiogram images for this report were reviewed by a single cardiologist (DY). Screening echocardiograms were obtained according to our institutional protocols at or before 36 weeks PMA or at transfer to SCH for patients with BPD. Timing of echocardiograms before transfer to SCH was determined by the referring hospital, but images from most hospitals were available for our review. Available echocardiograms performed at day of life 7–14 were reviewed for early PH (19). Echocardiograms with PH, including those diagnosed before 36 weeks PMA, were repeated within 1–4 weeks depending on severity of PH and right ventricular failure. Repeat echocardiogram could also be triggered by rising B-type natriuretic peptide (BNP) levels, which were obtained concomitantly and between echocardiograms. Serial echocardiograms were also performed to follow-up PDA, atrial septal defect (ASD), and pulmonary vein stenosis (PVS), at a frequency determined by the PH specialist and cardiologist. After echocardiogram showed resolution of PH, screening echocardiograms continued every 1–6 months, depending on age and degree of respiratory and oxygen support, until patients were off all respiratory support.

#### 2.2.4. PDA and ASD

Patients with a moderate or large PDA were recommended for cardiac catheterization for hemodynamic evaluation and potential closure by device at the time of BPD-PH diagnosis. Patients with moderate or large secundum ASD were recommended for device closure when BPD-PH did not resolve after optimizing respiratory support and treating aspiration. The devices used were the Amplatzer Piccolo Occluder, Medtronic Micro Vascular Plug and Siege Vascular Plug for PDA, and Amplatzer Septal Occluder device for ASD.

#### 2.2.5. PVS

Patients with evidence of PVS on echocardiogram underwent chest CT angiogram and cardiac catheterization evaluation with possible intervention.

#### 2.2.6. PH catheterization

Hemodynamic diagnostic catheterization before initiation of PH vasodilators, consistent with published recommendations (12), is standard practice at SCH. Patients who met the standard definition of PH, mean pulmonary artery pressure >20 mmHg, pulmonary capillary pressure <15 mmHg, and pulmonary vascular resistance >3 Woods units*m^2^ (20), were considered for PH medical therapy.

#### 2.2.7. PH medical therapy

Sildenafil was the first line pulmonary vasodilator after confirmation of PH by catheterization. Inhaled nitric oxide (iNO) was used before 36 weeks PMA in some mechanically ventilated infants with high FiO_2_ at SCH and referring hospitals prior to transfer, but due to difficulty verifying this data, iNO use was not collected. Bosentan and prostacyclins were not used in this cohort.

### 2.3. Data collected

Supplementary Table S1 lists collected data.

### 2.4. Statistical methods

**Figure 2:**
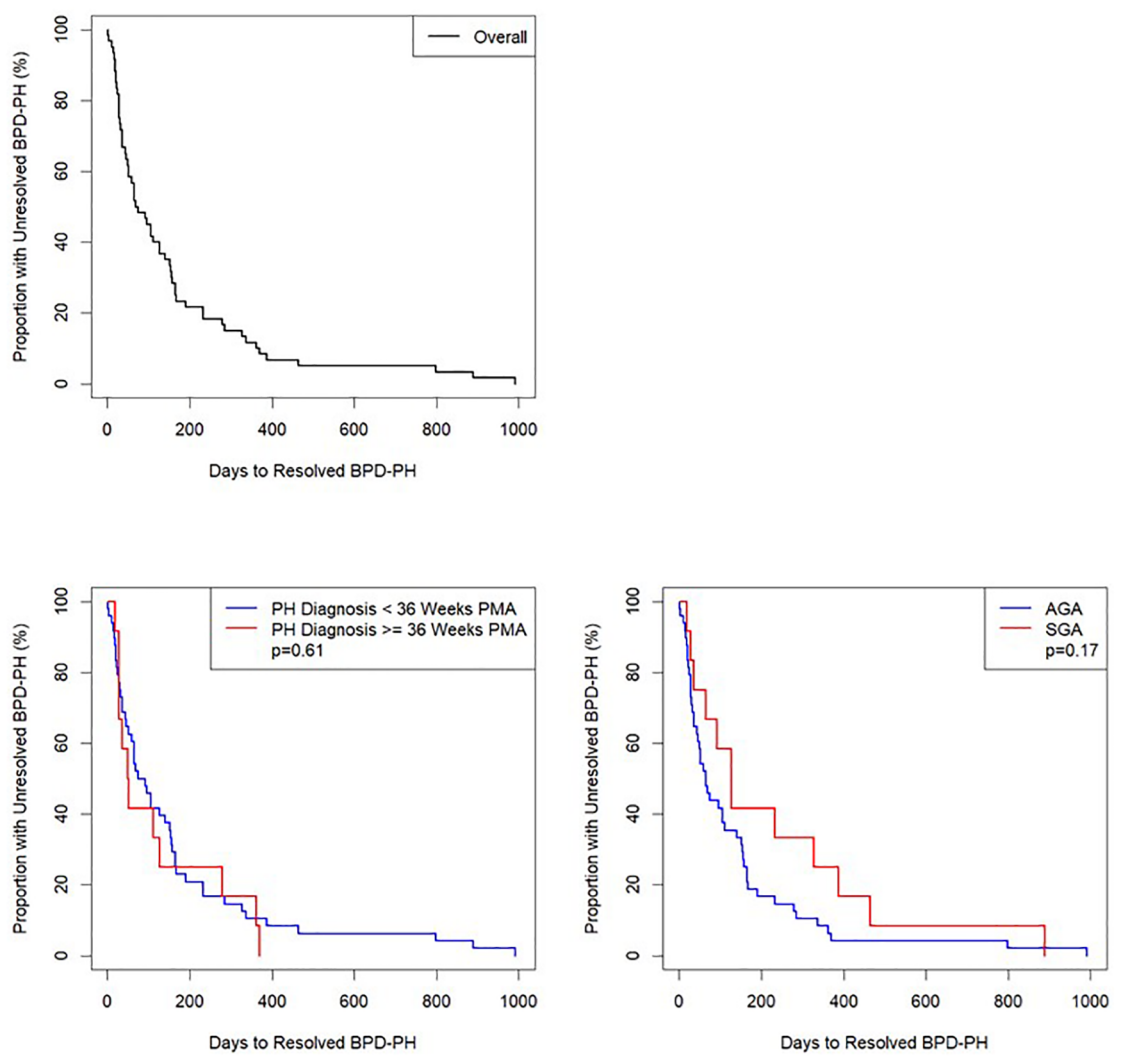
Kaplan–Meier curve of number of days from diagnosis to resolution of bronchopulmonary dysplasia-pulmonary hypertension (BPD-PH), *n* = 60. Top Left: entire cohort; Bottom Left: divided by BPD-PH diagnosed < or ≥ 36 weeks postmenstrual age (PMA); Bottom Right: divided by small for gestational age (SGA) or appropriate for gestational age (AGA). Log rank tests were calculated with significance defined as *p* < 0.05.

Analyses were conducted using R version 4.0.5. Descriptive statistics used for demographic and clinical characteristics included median/interquartile range (IQR) for continuous and counts/percentages for categorical variables. Length of time from BPD-PH diagnosis to resolution of BPD-PH was determined and presented as Kaplan-Meier plots (Figure 2). Potential outcomes of length of time to BPD-PH resolution were included in individual linear regression models with length of time to BPD-PH resolution as the predictor (Supplementary Table S5).

**Figure 3:**
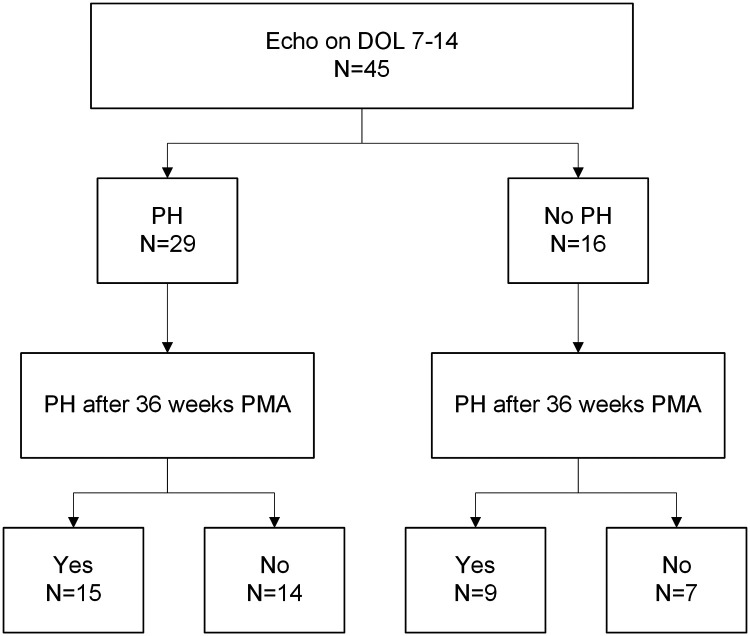
Flowchart of early echocardiogram PH diagnosis (DOL 7-14) and BPD-PH after 36 weeks PMA. Chi-squared test between groups has a *p*-value of 1. PH, pulmonary hypertension; BPD, bronchopulmonary dysplasia; DOL, day of life; PMA, postmenstrual age.

Log-rank tests (Figure 2) and individual linear regressions (Supplementary Table S6) were performed to test whether BPD-PH diagnosed before 36 weeks PMA (evolving BPD-PH) or small for gestational age (SGA) (21) status were associated with length of time to resolution of BPD-PH as predictors. A chi-squared test was used to test for association between echocardiographic evidence of PH on day of life 7–14 and >36 weeks PMA (Figure 3 and Supplementary Table S7). Missing data were excluded from all analyses, and no corrections for multiple testing were performed.

## 3. Results

### 3.1. Study cohort

A total of 91 infants had initial consultation by the multidisciplinary BPD team between 2018 and 2020. Ten patients with critical congenital heart disease (CHD) (Supplementary Table S2), defined as requiring surgery or catheter-based intervention in the first year of life (22), were excluded due to critical CHD being a major risk factor for death in prematurity (23–25) and the challenges defining whether PH was attributable to premature lung disease or CHD itself. Eleven patients who never had PH on echocardiogram were excluded. Ten patients who resolved BPD-PH before transfer to SCH were excluded due to lack of data and because the BPD team did not participate in management of BPD-PH. The final study cohort included 60 infants with BPD-PH (Supplementary Figure S1).

### 3.2. Characteristics of study cohort

Table 1 and Supplementary Table S3 list demographics and common comorbidities for the study cohort. All patients had follow-up through December 31, 2021, and inpatients were followed through September 2022. Two patients who died before discharge have missing data at discharge. The median gestational age was 24.9 weeks (IQR 24.1–26 weeks) and 20% of the cohort was SGA. Patients were transferred to SCH at a median of 5.4 weeks (IQR 1.7–13.2 weeks) after birth. Patients had initial consult by the BPD team at a median of 37.6 weeks (IQR 34.8–42.5 weeks) PMA. Twenty-one patients had initial consults before 36 weeks PMA, the earliest at 30.9 weeks. Almost half of the cohort (47%) met the definition of severe BPD type 2 (invasive ventilation at 36 weeks PMA), 25% underwent tracheostomy, and 42% went home on oxygen. At discharge, the vast majority (90%) were tube fed and 20% were post-pylorically fed.

**Table 1:** Demographic and clinical characteristics of patients with bronchopulmonary dysplasia-pulmonary hypertension (BPD-PH) (*n* = 60).

Variable	*N* (% of 60) or median (IQR)
Gestational age (weeks)	24.9 (24.1, 26)
Birthweight (g)	668 (590, 860)
SGA (20)^a^	12 (20%)
Male sex	39 (65%)
Race/Ethnicity (includes overlap)	
Any White	33 (55%)
Any Black	11 (18%)
Any Asian	8 (13%)
Any Hispanic	12 (20%)
Any Native American/Alaska Native	2 (3%)
Any other	2 (3%)
Declined to answer	2 (3%)
Age at transfer to center (weeks)	5.4 (1.7, 13.2)
PMA at transfer to center (weeks)	30.9 (26.6, 38.5)
PMA at diagnosis of BPD-PH (weeks)	28.1 (26.5, 33.4)
Died	3 (5%)
BPD Severity Type 2 (3)^b^	28 (47%)
ROP^c^	51 (85%)
IVH^c^	32 (53%)
NEC	21 (35%)
Invasive ventilation days	62 (34, 106)
Noninvasive positive pressure support days	80 (12, 128)
Missing data	9 (15%)
Tracheostomy	15 (25%)
Supplemental oxygen at discharge	25 (42%)
Missing data	2 (3%)
Feeding tube at discharge	54 (90%)
Missing data	2 (3%)
Post-pyloric feeds at discharge	12 (20%)
Missing data	2 (3%)

SGA, small for gestational age; PMA, postmenstrual age; ROP, retinopathy of prematurity; IVH, intraventricular hemorrhage; NEC, necrotizing enterocolitis.

^a^ See reference.

^b^ Severe BPD type 2 = invasive ventilation. See reference.

^c^ See Supplementary Table S3.

### 3.3. Deaths

There were three deaths, all after resolution of BPD-PH. Two patients died of complications of post-hemorrhagic hydrocephalus and ventriculoperitoneal shunt, at 11 and 21 months of age. One patient died after an airway emergency at home with tracheostomy change at 25 months of age.

### 3.4. PH diagnosis and resolution

The median (IQR) absolute age of first echocardiogram with PH was 2.5 (1.4, 8.2) weeks, and the median (IQR) PMA of first echocardiogram with PH was 28.1 (26.5, 33.4) weeks. BPD-PH was diagnosed before transfer to SCH in 29 (48%) patients. BPD-PH was diagnosed prior to 36 weeks PMA in 48 (80%) patients and after 36 weeks PMA in 12 (20%) patients.

BPD-PH resolved in all infants, including the three who died after resolution. The median (IQR) PMA of PH resolution was 43.5 (34.7, 55.8) weeks. BPD-PH resolved before 36 weeks PMA in 18 (30%) patients. In the group of 48 patients in whom BPD-PH was diagnosed before 36 weeks PMA, 30 continued to have BPD-PH after 36 weeks PMA. BPD-PH resolved between 36 and 52 weeks PMA in 24 (40%) patients, between 1 and 2 years of age in 15 (25%) patients, and greater than 2 years of age (specifically, 2.7, 3, and 3.3 years) in 3 (5%) patients. Thirteen (22%) had BPD-PH at hospital discharge.

The 26 patients who underwent cardiac catheterization for PDA closure resolved PH after a median (IQR) of 4 (0.3–16) weeks after PDA closure. Three patients who underwent ASD closure without PDA closure resolved PH after 1, 1, and 7 days.

Histograms showing PMA at BPD-PH diagnosis, BPD-PH resolution, and PDA or ASD closure are seen in Supplementary Figure S2.

### 3.5. PDA and ASD device closure

Twenty-four infants underwent successful cardiac catheterization for PDA closure alone, and one attempt was unsuccessful due to inferior vena cava clot and inability to establish access. Two patients underwent successful combined PDA and ASD closure and three underwent successful ASD closure alone. There were three catheterization complications during PDA closures: tricuspid valve damage in two patients and left pulmonary artery stenosis in one patient. There were no complications of ASD closure.

### 3.6. PH cardiac catheterization

Before eventual resolution of BPD-PH, three patients underwent diagnostic cardiac catheterization due to BPD-PH not improving by echocardiogram after respiratory optimization. Two met criteria for PH with mean pulmonary artery (PA) pressure >20 mmHg: 22 and 30 mmHg. In the third patient, catheterization revealed a significant shunt through a sinus venosus ASD as the cause of elevated mean PA pressure 21 mmHg, so the criteria for PH diagnoses was not met. Due to the location of the sinus venosus ASD, it had not been identified by echocardiogram and was not amenable to device closure. There were no diagnostic catheterization-related complications.

### 3.7. PH medication

Two patients diagnosed with BPD-PH by cardiac catheterization were started on sildenafil. The first patient had oxygen desaturation requiring an increase in the amount of supplemental oxygen to maintain oxygen saturation above 94% for 2–6 h following administration, resulting in discontinuation after the 4th dose. The second patient had urticaria and hypotension requiring treatment for anaphylaxis 21 days after initiation of sildenafil, and it was discontinued. BPD-PH resolved in both patients after discontinuation of sildenafil, the first at 58 weeks PMA and the second at 3 years of age.

Three additional patients were started on sildenafil without cardiac catheterization. One patient transferred to SCH at PMA 40.9 weeks and echocardiogram showed tricuspid regurgitation jet velocity 2.8 m/s; due to clinical instability iNO was transitioned to sildenafil at 1 mg/kg TID without cardiac catheterization data. PH resolved at 57 weeks PMA and the patient was allowed to outgrow sildenafil until the dose reached 0.5 mg/kg, after 41 weeks of treatment. Two other patients were transferred to SCH already on sildenafil. PH resolved after optimization of respiratory support, and sildenafil was stopped after the dose was outgrown to 0.5 mg/kg/dose after 15 and 36 weeks of therapy (Supplementary Table S4).

### 3.8. Outcomes and predictors of time to PH resolution

Time to PH resolution was not significantly associated with queried clinical outcomes (Supplementary Table S5). Neither PH diagnosis <36 or ≥36 weeks PMA nor SGA status had significant individual associations as predictors of time to PH resolution (Figure 2, Supplementary Figure S2, and Supplementary Table S6)

### 3.9. PVS

Two patients underwent cardiac catheterization for PVS treatment. One patient developed PVS of the left upper, left lower, and right upper pulmonary veins at 15 months of age, in the setting of BPD-PH, and PH resolved by echocardiogram the day following PVS treatment. There have been multiple subsequent PVS interventions. The second patient developed PVS in the left upper pulmonary vein at 52 weeks PMA without associated PH after resolution of BPD-PH and did not have recurrence of PH or PVS after one intervention.

### 3.10. Early echocardiogram prediction of PH

To assess whether early echocardiograms at DOL 7 are associated with PH at 36 weeks in our cohort, we looked at the entire 91-patient cohort of severe BPD to include patients that never had PH. Of these patients, 45 had echocardiograms performed between day of life 7–14. We compared these to echocardiograms in the same patients at 36 weeks PMA for presence of BPD-PH (Figure 3 and Supplementary Table S7). The chi-squared test *p*-value was 1, indicating no significant association between BPD-PH diagnosis in early and later echocardiograms.

## 4. Discussion

### 4.1. Principal findings

#### 4.1.1. BPD-PH diagnosis and resolution

Our cohort of patients with severe BPD all had resolution of BPD-PH with a strategy of multidisciplinary team care including optimization of respiratory support and interventional cardiac catheterization device closure of PDA and ASD, with limited use of sildenafil (8%) and low mortality (5%). To treat BPD-PH, we employed BPD-specific ventilator strategies, including low rate, higher tidal volume, longer inspiratory time, and PEEP titrated to treat multi-level airway disease (4). A quarter of the patients in our cohort required tracheostomy. Unlike respiratory strategies for younger premature infants, only mild hypercapnia was permitted with target pCO_2_ less than 55 mmHg, and oxygen was added to achieve goal saturations above 94%. Respiratory support was considered optimized if infants demonstrated acceptable growth, minimal work of breathing, and could participate in developmental therapies. Outpatients with improving BPD-PH on respiratory support continued echocardiogram screening until BPD-PH eventually resolved, some taking over 2 years. Our team was also aggressive in treating potential aspiration as recommended by PH experts (26), with 90% of study patients discharged with a feeding tube and 20% on post-pyloric feeds.

BPD-PH was diagnosed relatively early, with half diagnosed before 2.5 weeks of life and 28.1 weeks PMA, and 48% diagnosed before transfer to SCH. Resolution occurred before 36 weeks PMA for 30%, between 36 and 52 weeks PMA for 40%, and after 52 weeks for 30%. In this large series of infants who received aggressive care for PDAs, BPD-PH resolution occurred a median of 4 weeks after device closure of moderate or large PDA. We note disproportionate representation of SGA in our cohort (20%), consistent with publications identifying SGA as a risk factor for development of PH in patients with BPD (27). However, despite being implicated as risk factors for severity of disease (19, 27), neither SGA nor PH diagnosis at PMA < 36 weeks were associated with length of time to PH resolution, suggesting that our multidisciplinary care may have negated some of the risks associated with these factors.

These results are similar to a cohort reported by Altit et al. in which the “natural history” of BPD-PH was resolution, although their population was solely those with BPD-PH diagnosed after 36 weeks, with markers of more severe BPD, over a time period of nearly two decades (28). However, our cohort had lower mortality (5%) with the rare deaths unrelated to BPD-PH or heart failure. While we expect one result of our team-based approach to be lower mortality, the retrospective nature of this study and lack of an adequate historical control group makes it impossible to confirm causality. We hope with prospective data collection and improved guidelines for timing of referral for patients with severe BPD that we will be better able to address the impact of our multidisciplinary team in the future through single and multicenter studies.

#### 4.1.2. High rates of intervention for PDA and ASD

Cardiology management played an important role in our management of BPD-PH, with almost half of the patients undergoing PDA or ASD device closure. Assessment and closure of both PDA and ASD were supported by recent findings that the presence of a PDA and an ASD is associated with increased risk of BPD-PH over time (29, 30). We used PDA and ASD size, not hemodynamic significance, as the indication for closure. This is because while a large PDA is not hemodynamically significant if there is high PVR, a large PDA will transmit systemic pressure to the pulmonary arteries. In addition, an ASD that causes even mild pulmonary over-circulation can lead to respiratory compromise in patients with lung dysplasia (30, 31). Historically, PDAs were surgically ligated, but newer devices make PDA and ASD closure in the cardiac catheterization lab an option with an acceptable level of risk in even very small infants. Likewise, while ASDs have not previously been considered important to the development of PH in neonates or infants and are generally recommended for closure in childhood before school age, the ability to safely close ASD in infants at SCH influenced our management recommendations and may have contributed to rapid resolution of BPD-PH. Contrary to conventional teaching to leave PDA or ASD as a “pop-off” in the setting of low velocity or bidirectional shunting, there has been no instance of right ventricular failure after closure in our cohort. This practice has been developed with the interventional cardiology team at our center, with whom we are fortunate to have a close collaboration.

#### 4.1.3. Low sildenafil use

Our results show much lower use of sildenafil in the BPD-PH population (8%) than in other reports, including the multicenter Children's Hospital Neonatal Consortium, in which sildenafil use averaged 60% per center (11). We suspect our lower sildenafil use was due to several factors. First, the presence of BPD-PH on the echocardiogram signaled that respiratory support was sub-optimal and would guide changes in multidisciplinary team recommendations. Also, instead of initiating sildenafil when the echocardiogram continued to show BPD-PH after respiratory optimization, we elected to watch and reassess if the echocardiogram demonstrated improvement in BPD-PH. In addition, by having a cardiology-trained BPD team member comparing echocardiogram images, subtle improvements in BPD-PH not noted in the official echocardiogram report could be observed. Lastly, we are committed to diagnosing PH by cardiac catheterization, for reasons as discussed in 4.1.4. below.

Furthermore, we speculate that even the three patients on long-term sildenafil may have resolved PH without treatment. We noted that the two patients who stopped sildenafil early due to adverse events and still eventually resolved BPD-PH were clinically similar to the third patient who was started on sildenafil by our group. In addition, BPD-PH resolved after involvement of our care in the two patients who were started on sildenafil by the referring hospital.

Whether higher use of sildenafil without diagnostic cardiac catheterization would have led to even earlier resolution of BPD-PH is unknown. However, increased sildenafil exposure would have increased risk of adverse events. The two adverse events that led to stopping sildenafil were well-documented and agreed upon by all team members. Hypoxemia in one patient after initiation of sildenafil may be due to lack of hypoxic vasoconstriction, leading to ventilation—perfusion mismatching, as others have speculated in BPD (32). Although many reports have demonstrated sildenafil safety in neonates (18, 33), we share concerns for unintended impacts of sildenafil on vascular development, such as those raised after sildenafil-exposed fetuses demonstrated increased risk of neonatal PH (34). We look forward to the results of a placebo-controlled clinical trial studying safety of sildenafil in BPD that is currently under way (35).

#### 4.1.4. Cardiac catheterization diagnosis of PH

While our practice is to perform cardiac catheterization to diagnose PH, recently published work supports the empiric initiation of sildenafil without cardiac catheterization due to catheterization-associated risks, lack of clinical utility, and cost (17). Because we perform fewer cardiac catheterizations and initiate sildenafil less frequently, diagnostic cardiac catheterization before sildenafil initiation appears to have a beneficial role in our practice and patient population. This practice also allows for identification and treatment of other cardiac pathologies such as hemodynamically significant sinus venosus ASD as identified in one of our patients.

#### 4.1.5. Echocardiogram screening of PH

Standards for timing and interpretation of screening echocardiogram for BPD-PH or elevated pulmonary pressures associated with evolving BPD remain unclear (36, 37). However, echocardiogram screening for PH before 36 weeks may influence early approaches to mitigate PH, such as ventilator strategies, PDA/ASD closure, and avoidance of aspiration. In our cohort, PH at DOL 7-14 echocardiogram was not associated with late PH at 36 weeks PMA, in contrast to findings from Mourani et al. (19). However, we acknowledge that our contemporary cohort is notably smaller with variable timing of echocardiograms and therefore may not provide accurate representation of early PH in the evolving BPD population across other centers.

### 4.2. Strengths

This is a contemporary cohort treated after publication of BPD-PH management guidelines (4, 12, 15, 38). Our population of patients with severe BPD was comparable to other published level IV NICU cohorts with respect to severity of BPD, gestational age, birth weight, and complications of prematurity such as IVH, NEC, and ROP, with a high level of acuity as a multistate regional referral center (39). Patient selection for inclusion in BPD rounds was objectively defined. Patients were treated by the BPD team in a systematic way, starting with screening echocardiograms, diagnosis, and treatment of BPD-PH. Echocardiogram images were reviewed by a consistent reader (DY), addressing the limitations of this modality and potential for subjectivity. Follow-up included echocardiography at least 1 year out in all infants, including evaluation beyond resolution.

### 4.3. Limitations

We acknowledge limitations of this retrospective review. Our analysis may provide insight but cannot confirm causality. Specifics of respiratory support optimization are not further delineated but were titrated to varying BPD phenotypes and associated physiologies (40). We relied heavily on the combined expertise of our specific subspecialties in fine-tuning respiratory support, including significant contributions of dedicated respiratory therapists. We were not involved in treatment before transfer to SCH and did not reliably have access to details of prior management or prior echocardiograms. Diagnosis of PH by echocardiogram remains somewhat subjective, and in cases without measurable tricuspid regurgitation, may be overestimated if using flattening of the interventricular septum. Hemodynamics were not assessed at cardiac catheterization for most patients who were catheterized specifically for device closure. We did not describe BNP trends due to an excess of values for assessment. Use of postnatal steroids, diuretics, and inhaled beta agonists was not evaluated. Due to this cohort starting in 2018 with follow-up through 2021, we are not able to evaluate if there are any patients who present as older children or adults with PH (41, 42). Small sample size may limit power to identify predictors and outcomes of time to PH resolution.

### 4.4. Clinical relevance

BPD-PH managed by a multi-disciplinary team, including optimization of respiratory support and early closure of PDA and ASD, may lead to improved mortality and resolution of BPD-PH, with low rates of pulmonary vasodilator use. Comparable time to resolution in patients anticipated to have persistent disease due to early diagnosis and/or SGA status supports this approach. Continuity of team members was key to recognizing subtle improvement, or lack thereof, and to tracking progress over time. Input from bedside nurses, respiratory therapists, nutritionists, physical therapists, occupational therapists, and feeding specialists was also essential to care. While optimizing respiratory status may lead to longer time on more invasive respiratory support, it likely allows for BPD recovery with both somatic and lung growth. In addition to following clinical progress, frequent screening echocardiograms evaluating the trajectory of BPD-PH should be used to guide whether respiratory support is optimized. Based on our lower usage of sildenafil, hemodynamic cardiac catheterization before initiation of sildenafil remained useful to confirm diagnosis of BPD-PH. While sildenafil remains first line medical therapy (33) for BPD-PH, we think it is prudent to await the results of ongoing and future research to best guide usage.

## Data Availability

The raw data supporting the conclusions of this article will be made available by the authors, without undue reservation.
