# Supplementary material for: A multidisciplinary approach to severe bronchopulmonary dysplasia is associated with resolution of pulmonary hypertension
Source: Front Pediatr. 2023 Mar 30;11:1077422. doi: 10.3389/fped.2023.1077422 (PMC10098720; doi:10.3389/fped.2023.1077422)
Supplement: Supplementary file 1 [file Datasheet1.docx]

Supplemental Tables and Figures

Supplementary Table 1. Collected Data.

| **Demographics** | - Gestational age - Birthweight - Small for Gestational Age status (1) - Sex - Race - Age at transfer to Seattle Children’s Hospital (SCH) - Death |
| --- | --- |
| **Bronchopulmonary Dysplasia (BPD) Severity** defined by the BPD Collaborative at 36 weeks Postmenstrual Age (PMA) (2) | - Severity Type 1 (continuous positive airway pressure [CPAP], high-flow nasal cannula [HFNC], and ≥ 30% oxygen) - Severity Type 2 (invasive ventilation) |
| **Common Neonatal Intensive Care Unit (NICU) morbidities** | - Retinopathy of prematurity (ROP) - Interventricular hemorrhage (IVH) - Necrotizing enterocolitis (NEC) |
| **Respiratory Support** | - Days of invasive ventilation - Days of non-invasive positive pressure support - Tracheostomy - Supplemental oxygen at discharge |
| **Feeding** | - Feeding tube at discharge - Post-pyloric feeding at discharge |
| **Echocardiogram evidence of pulmonary hypertension (PH)** at the following timepoints: | - 7 – 14 days of age - First echocardiogram to show pulmonary hypertension - First echo to show resolution of BPD-PH - Subsequent screening echo after resolution with recurrence of PH or pulmonary vein stenosis |
| **Cardiac catheterization** | - Indication: Closure of Patent Ductus Arteriosus (PDA) or Atrial Septal Defect (ASD) - Hemodynamics - Complications |
| **Pulmonary Vasodilator Therapy** | - Medication and dose - Timing of initiation and discontinuation (sildenafil) |

Supplementary Table 2. Critical Congenital Heart Disease diagnoses in the BPD cohort.

| **Critical Congenital Heart Disease** |
| --- |
| Transposition of the great vessels, interrupted aortic arch |
| Partial atrioventricular septal defect, critical coarctation |
| Severe stenosis of all pulmonary veins due to RASA1 mutation |
| Tetralogy of Fallot (x2) |
| Pulmonary atresia with ventricular septal defect |
| Severe coarctation and hypoplastic mitral valve |
| Pulmonary atresia with intact ventricular septum |
| Critical pulmonary valve stenosis |
| Complete atrioventricular septal defect |

BPD = bronchopulmonary dysplasia. RASA1 = RAS P21 Protein Activator 1.

Supplementary Table 3. Common Comorbitities by Stage and Grade.

| ROP Comorbidities Stage |  |
| --- | --- |
| 0, N (%) | 2 (3%) |
| 1, N (%) | 14 (23%) |
| 2, N (%) | 17 (28%) |
| 3, N (%) | 17 (28%) |
| 4, N (%) | 1 (2%) |
| No ROP Comorbidities, N (%) | 9 (15%) |
| IVH Comorbidities Grade |  |
| I, N (%) | 9 (15%) |
| II, N (%) | 6 (10%) |
| III, N (%) | 1 (2%) |
| IV, N (%) | 16 (27%) |
| No IVH Comorbidities, N (%) | 28 (47%) |

Supplementary Table 4. Sildenafil Use in Five Patients.

| Reason to start sildenafil | Gestational age at birth | PMA (weeks) at sildenafil start | PMA (weeks) at PH resolution | Weeks from sildenafil start to PH resolution | Length of sildenafil therapy | Reason to stop sildenafil |
| --- | --- | --- | --- | --- | --- | --- |
| Mean PAp 30 | 29 1/7 | 50 3/7 | 57 6/7 | 7 3/7 | 1 day | desaturation |
| Mean PAp 22 | 25 2/7 | 66 6/7 | 154 3/7 | 87 4/7 | 21 days | anaphylaxis |
| Started at referring hospital | 24 2/7 | 33 | 55 2/7 | 22 2/7 | 115 days  (16.4 weeks) | PH resolved and outgrew dose |
| Started at referring hospital | 25 5/7 | 41 5/7 | 63 2/7 | 21 4/7 | 249 days  (35.6 weeks) | PH resolved and outgrew dose |
| Echo TR 2.8 m/s | 24 2/7 | 45 5/7 | 54 3/7 | 9 5/7 | 290 days  (41.4 weeks) | PH resolved and outgrew dose |

PMA = postmenstrual age. PH = pulmonary hypertension. PAp = pulmonary artery pressure. TR = tricuspid regurgitation.

Supplementary Table 5. Linear regression associations of time from PH diagnosis to resolution with various binary and continuous outcomes. Coefficients are interpreted as the average difference in the outcome measure comparing a group with 1 day longer time to PH resolution to a group with 1 day shorter time to PH resolution.

| Outcome (Binary or Continuous) | n  (of 60) | Time to PH Resolution Coefficient (Robust 95% CI) | Robust p-value |
| --- | --- | --- | --- |
| Post-Pyloric Feeds at Discharge‡ | 58 | -2e-04 (-5e-04, 1e-04) | 0.1983 |
| Feeding Tube at Discharge‡ | 58 | 2e-04 (0, 3e-04) | 0.0663 |
| Tracheostomy‡ | 60 | 3e-04 (-3e-04, 8e-04) | 0.3778 |
| Invasive Ventilation Days† | 60 | 0.0072 (-0.2114, 0.2259) | 0.9482 |
| Noninvasive Positive Pressure Ventilation Days† | 51 | 0.0357 (-0.1578, 0.2291) | 0.7178 |
| Severity of BPD (Type 2)* ‡ | 60 | -1e-04 (-7e-04, 6e-04) | 0.8402 |
| Supplemental Oxygen at Discharge‡ | 58 | -1e-04 (-7e-04, 5e-04) | 0.8162 |

PH = pulmonary hypertension. BPD = bronchopulmonary dysplasia. ‡ Binary outcome. † Continuous outcome.

*Severe BPD type 1 = continuous positive airway pressure (CPAP), high-flow nasal cannula (HFNC), and ≥ 30% oxygen; type 2 = invasive ventilation. See reference (1).

Supplementary Table 6. Individual linear regression associations of binary predictors PH diagnosis ≥ 36 weeks PMA and small for gestational age with days from PH diagnosis to resolution. Coefficients are interpreted as the average difference in the days to PH resolution comparing a group with the predictor to a group without the predictor.

| Binary Predictor of Days to PH Resolution | n (of 60) | Coefficient (Robust 95% CI) | Robust p-value |
| --- | --- | --- | --- |
| PH Diagnosis ≥ 36 Weeks PMA | 60 | -37.94 (-133.15, 57.27) | 0.4348 |
| SGA* | 60 | 97.79 (-50.1, 245.68) | 0.195 |

PH = pulmonary hypertension. SGA = small for gestational age. *As defined in reference (2).

Supplementary Table 7. Cross-tabulation of PH on DOL 7 echo and PH at 36 weeks PMA. Chi-squared test between groups has a p-value of 1.

|  | PH at 36 weeks PMA | No PH at 36 weeks PMA | Total |
| --- | --- | --- | --- |
| PH on DOL 7 echo | 15 | 14 | 29 |
| No PH on DOL 7 echo | 9 | 7 | 16 |

DOL = day of life. PH = pulmonary hypertension. PMA = post menstrual age.

Supplementary Figure 1. Consort diagram of cohort.

PH = pulmonary hypertension.

Supplementary Figure 2. Histograms of postmenstrual age (PMA) at (A) Bronchopulmonary Dysplasia-Pulmonary Hypertension (BPD-PH) Diagnosis, n=60, (B) BPD-PH Resolution, n=60, and (C) Patent Ductus Arteriosus (PDA) or Atrial Septal Defect (ASD) closure by cath, n=30.

A.


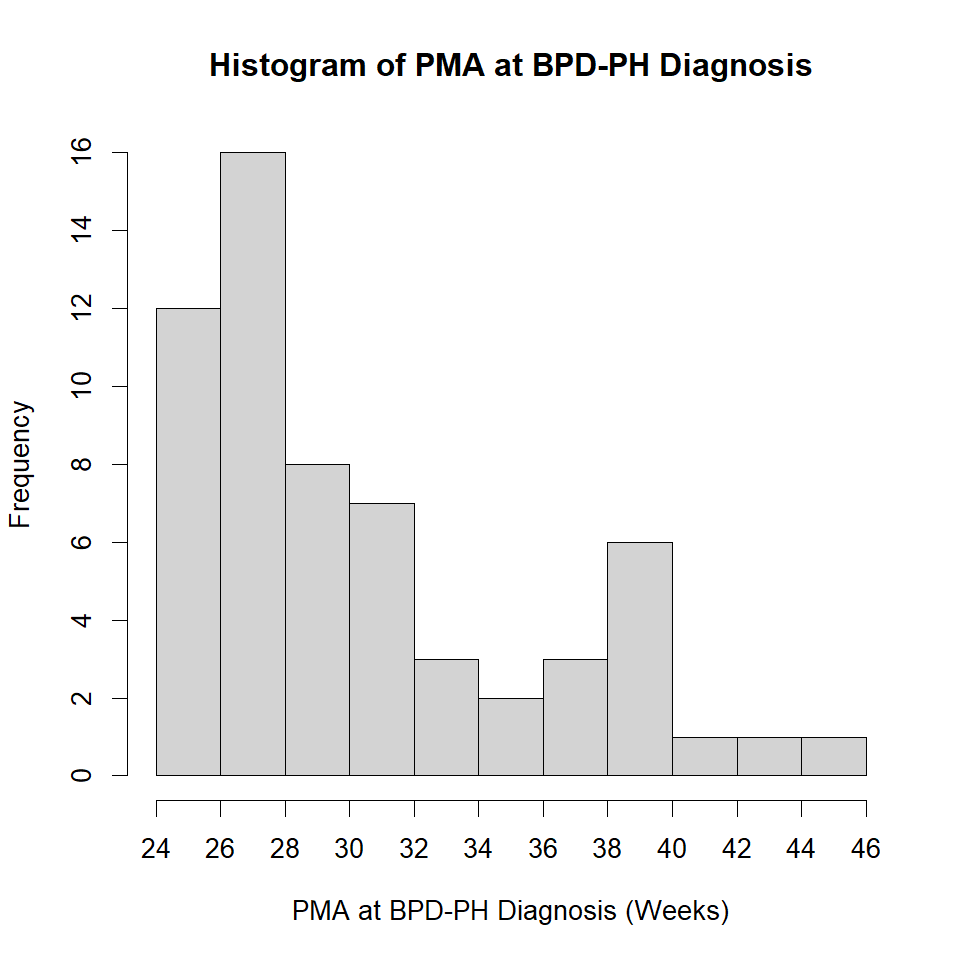


B.


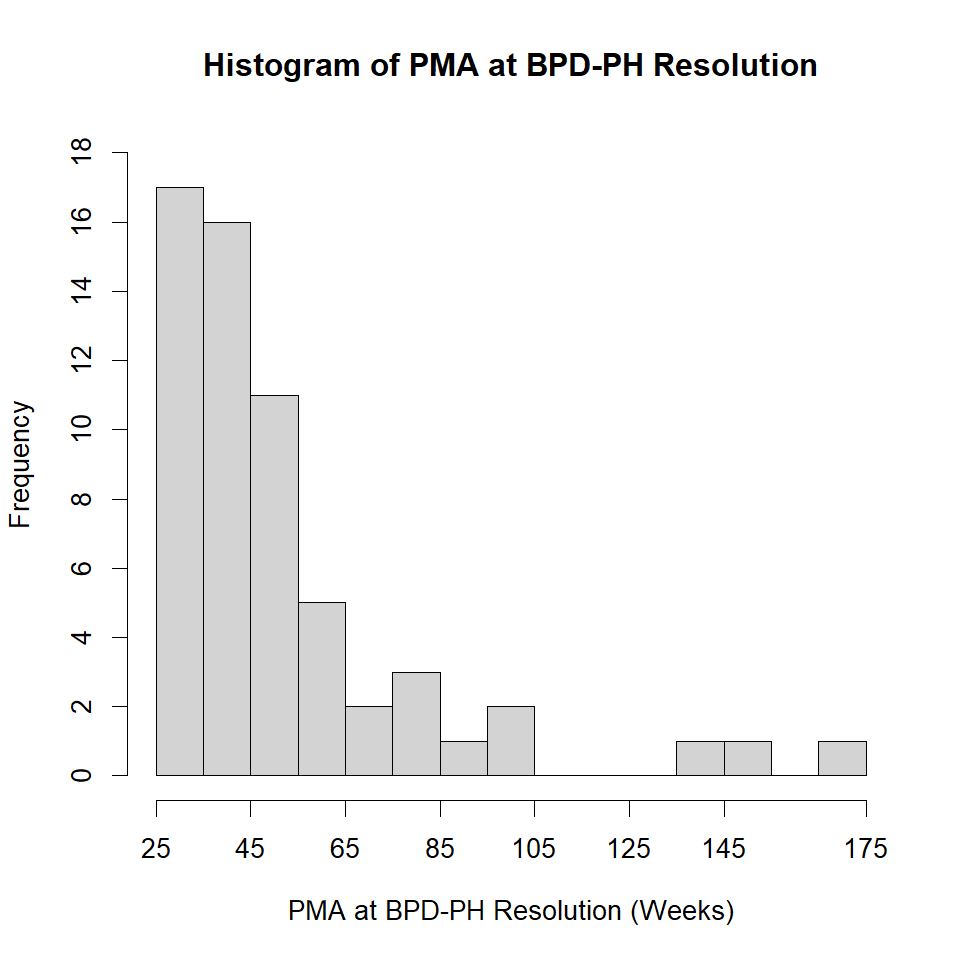


C.


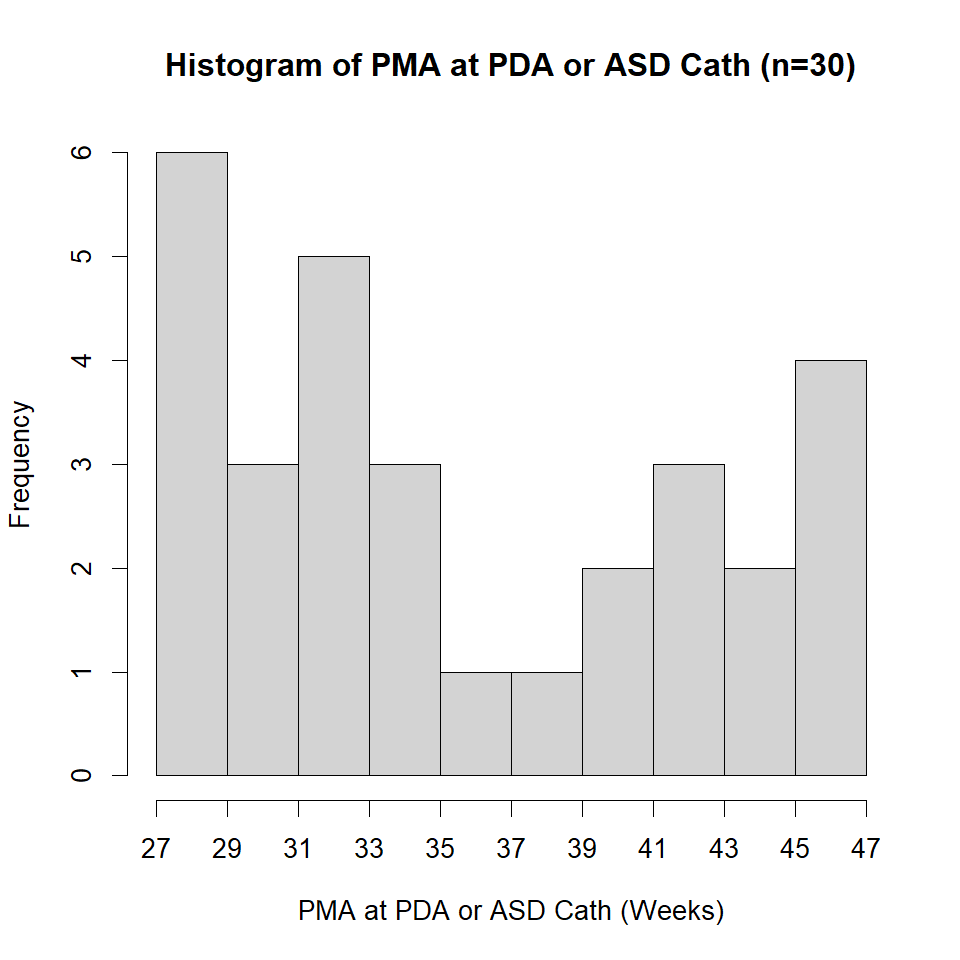


**References**

1. Fenton TR, Kim JH. A systematic review and meta-analysis to revise the Fenton growth chart for preterm infants. BMC Pediatr. 2013;13:59.

2. Abman SH, Collaco JM, Shepherd EG, Keszler M, Cuevas-Guaman M, Welty SE, et al. Interdisciplinary Care of Children with Severe Bronchopulmonary Dysplasia. J Pediatr. 2017;181:12-28 e1.
